# Characteristic electron-microscopic features of cryofibrinogen-associated glomerulonephritis: a case report

**DOI:** 10.1186/s12882-020-1696-0

**Published:** 2020-01-29

**Authors:** Emi Ibuki, Aiko Shiraishi, Tadashi Sofue, Yoshio Kushida, Kyuichi Kadota, Kazuho Honda, Dedong Kang, Kensuke Joh, Tetsuo Minamino, Reiji Haba

**Affiliations:** 1 Department of Diagnostic Pathology, Kagawa University, 1750-1 Ikenobe, Miki-cho, Kita-gun, Kagawa, 761-0793 Japan; 2 Division of Nephrology and Dialysis, Department of Cardiorenal and Cerebrovascular Medicine, Kagawa University, Kagawa, Japan; 3 Division of Microscopic Anatomy, Department of Anatomy, Showa University School of Medicine, Tokyo, Japan; 4 Department of Pathology, Jikei University School of Medicine, Tokyo, Japan

**Keywords:** Cryofibrinogen, Cryofibrinogen-associated glomerulonephritis, Membranoproliferative glomerulonephritis, Organized deposit, Microtubular structure

## Abstract

**Background:**

Cryofibrinogenemia is a rare disorder that mainly affects the skin and occasionally the kidney. However, there are few published reports of cryofibrinogenemia-associated renal pathology. We therefore report a patient with cryofibrinogen-associated glomerulonephritis. Samples from this patient were examined by electron microscopy, laser microdissection, and liquid chromatography-tandem mass spectrometry (LC-MS/MS).

**Case presentation:**

A 78-year-old Japanese man presented with declining renal function, proteinuria, and gross hematuria. Kidney biopsy showed a membranoproliferative pattern with crescent formation and dominant C3c deposition in which subendothelial deposits with uniquely organized electron-microscopic features were observed. Additional ultrastructural analysis of cryoprecipitates extracted from plasma revealed similar structures of the glomerular subendothelial deposits. LC-MS/MS identified an increase in fibrinogen α, β, and γ chains, fibronectin, filamin-A, and C3. The glomerular lesions were diagnosed as cryofibrinogen-associated glomerulonephritis on the basis of these findings.

**Conclusions:**

Although there are few reports of cryofibrinogen-associated glomerulonephritis, we believe that accurate diagnosis can be achieved by performing LC-MS/MS and ultrastructural analysis.

## Background

Cryofibrinogen is an abnormal protein that precipitates when plasma is stored at 4 °C and redissolves at 37 °C [1]. Cool temperature-induced precipitation of proteins including fibrinogen, in plasma but not in serum, enables differentiation of cryofibrinogens and cryoglobulins. Cryoprecipitates from plasma consist of fibrinogen, fibrin, fibronectin, factor VIII, and smaller amounts of various plasma proteins [2], whereas cryoprecipitates from serum consist mainly of cryoglobulins. Cryofibrinogenemia may be primary or secondary to malignancies, infections, autoimmune diseases, thromboembolic diseases, or other processes. Clinical manifestations of cryofibrinogenemia vary from no symptoms to thromboembolic phenomena. The skin is usually the target organ: manifestations including purpura, livedo reticularis, Raynaud’s phenomenon, ulceration, and gangrene [3]. Although the kidney can also be a target organ, there are few reports describing the renal pathology of cryofibrinogenemia-related glomerulopathy [4–7]. Sethi et al. described two cases [6] and Sudo et al. one case of cryofibrinogen-associated glomerulonephritis [7]. Organized deposits were observed in the subendothelial area in all three cases. In addition, in one case reported by Sethi et al. and in the case described in this report, deposits found in the kidney biopsy specimen and cryoprecipitates extracted from the patient’s plasma had identical ultrastructural features.

In this report, we describe a case of cryofibrinogen-associated glomerulonephritis in which electron microscopic findings, laser microdissection, and liquid chromatography-tandem mass spectrometry (LC-MS/MS) were useful for diagnosis.

## Case presentation

A 78-year-old Japanese man presented with declining renal function, proteinuria, and hematuria. He had started to experience gross hematuria 7 months before admission. Examination by the Urology Department revealed no evidence of urinary tract neoplasia. He was therefore transferred to the Nephrology Department.

His medical history included hepatitis B virus and hepatitis C virus carrier status with an undetectable viral titer, gastric carcinoma (post-surgery), cerebral infarction, hypertension, paroxysmal atrial fibrillation, and non-tuberculous mycobacterial infection. The patient had no history of cutaneous rashes or ulcers. He was under medication with apixaban, amlodipine, losartan and febuxostat. However, to perform a kidney biopsy, apixaban was discontinued at his first visit to the Nephrology Department.

Laboratory investigations at the time of kidney biopsy are shown in Table 1. A serum creatinine concentration of 3.51 mg/dL corresponded to an estimated glomerular filtration rate (eGFR) of 14 mL/min/1.73m^2^ (revised equations for estimated GFR from serum creatinine in Japan [8]). The patient had anemia with a hemoglobin concentration of 7.9 g/dL. White blood cells, platelet counts and liver function were within normal ranges. C-reactive protein was 0.33 mg/dL. Serologic testing for antineutrophil cytoplasmic antibodies, antinuclear antibodies, and cryoglobulins were all negative, and C3, C4 and CH50 titers were within the reference ranges. Urinalysis showed protein (4+) and blood (3+), and 24-h urinary protein was 4.5 g. Serum and urine electrophoresis and immunofixation showed the presence of an IgG-κ band (serum IgG 1.177 mg/dL, κ/λ ratio 2.8). However, no proliferation of monoclonal plasma cells was detected in a bone marrow aspirate (only 3.2% plasma cells). Accordingly, a diagnosis of IgG-κ type monoclonal gammopathy of unknown significance was made. Additionally, computed tomography scanning revealed no evidence of malignancy.

**Table 1:** Laboratory data at the time of kidney biopsy

Data	Value	Units	Upper limit	Lower limit
White blood cells	7600	/μL	8700	4700
Red blood cells	258	× 10^4^/μL	540	400
Hemoglobin	7.9	g/dl	17.0	13.0
Hematocrit	23.6	%	50.0	40.0
Platelets	27.2	×10^4^/μL	35.0	15.0
C-reactive protein (CRP)	0.33	mg/dl	0.20	0.00
Total protein	5.8	g/dl	8.2	6.5
Albumin	2.5	g/dl	5.5	3.5
Total bilirubin	0.3	mg/dl	1.2	0.1
Alkaline phosphatase (ALP)	202	IU/L	340	100
Aspartate aminotransferase (AST)	35	IU/L	35	10
Alanine aminotransferase (ALT)	15	IU/L	40	5
Lactate dehydrogenase (LDH)	259	IU/L	220	110
γ-glutamyl transpeptidase (GTP)	14	IU/L	60	0
blood urea nitrogen (BUN)	55.6	mg/dl	20	7
Creatinine	3.51	mg/dl	1.30	0.70
Uric acid	5.0	mg/dl	8.2	4.3
eGFR	14.0	ml/min		
Sodium	142	mEq/L	146	135
Potassium	4.5	mEq/L	4.5	3.5
Chloride	106	mEq/L	110	96
Calcium	8.1	mg/dl	10.2	8.2
triglyceride	168	mg/dl	149	30
HDL cholesterol	58	mg/dl	75	40
LDL cholesterol	120	mg/dl	130	70
Glycosylated hemoglobin (HbA1c)		%	6.2	4.6
Immunoglobulin G (IgG)	1177	mg/dL	435	114
Immunoglobulin A (IgA)	203	mg/dL	1700	870
Immunoglobulin M (IgM)	36	mg/dL	190	33
C3	83	mg/dL	144	68
C4	30	mg/dL	33	12
Complement activities (CH50)	52.7		50	30
Antinuclear antibody	< 40	Fold	40	0
PR3-ANCA	< 3.0	U/ml	3.5	0
MPO-ANCA	< 3.5	U/ml	3.5	0
Cryoglobulin	Negative			
Ferritin	528	ng/ml	465	39
Prothrombin time (PT) INR	0.93		1.15	0.85
Activated partial thromboplastin time (APTT)	22.7	sec	40	27
D-dimer	3.1	μg/ml	1.0	0.0
Hepatitis B surface antigen	Negative			
Anti-hepatitis B surface antigen	Positive			
Anti-hepatitis B core antigen	Positive			
HBV-DNA	Undetectable	LogU/ml		
HCV antibody	Positive			
HCV-RNA	Undetectable			
Serum electrophoresis	IgG-κ band			
κ/λ ratio	2.8	Fold	1.804	0.248
Urinary occult blood (dipstick)	3+			
Urinary protein (dipstick)	4+			
Urinary protein	4.5	g/day	0.15	0
Urinary NAG	81.4	U/L	11.2	0.7
Urinary β2 macroglobulin	5719	μg/L	250	0
Urinary Bence Jones protein	IgG-κ band			

**Fig. 1:**
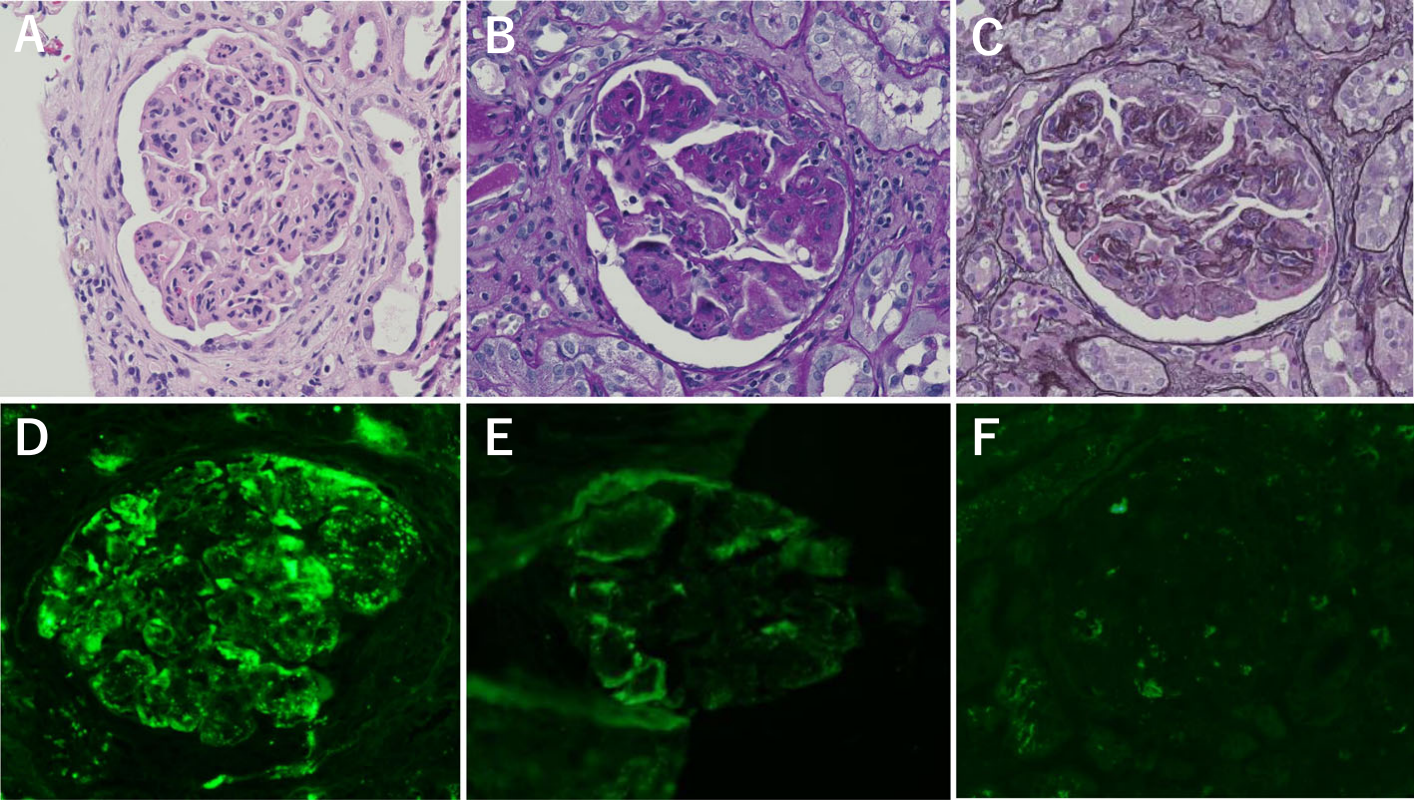
Light microscopy and immunofluorescence microscopy findings. **a**–**c** Membranoproliferative glomerulonephritis with crescent formation. Neutrophils are present in the capillary lumens. (**a**: hematoxylin and eosin stain, **b**: periodic acid-Schiff stain, **c**: periodic acid methenamine silver stain); **d**–**f** staining for C3 (**d**), IgM (**e**) and weak staining for fibrinogen (**f**). (**a**–**e**: original magnification, 40×)

Fifteen glomeruli were identified in a renal biopsy, three of which showed global sclerosis under light microscopy. The remaining glomeruli showed lobular accentuation of the glomerular capillary tufts with diffuse mesangial expansion. Endocapillary proliferation with neutrophilic infiltration was noted. Peripheral capillary walls were thickened and had double contours. Subendothelial deposits negative for periodic acid methenamine silver staining and positive for periodic acid Schiff (PAS) staining were observed. Cellular crescents were also present in six glomeruli. There was moderate interstitial fibrosis and tubular atrophy (50%) (Fig. 1a–c). There were no remarkable changes in vessels.

Immunofluorescence studies showed that C3c was dominantly positive between the capillary walls and mesangial area. Capillary walls were also positive for IgM and regions of the mesangial area were weakly positive for fibrinogen. IgG, IgA, C1q, kappa and lambda light chains were all negative (Fig. 1d–f).

Ultrastructural examination revealed the presence of unique subendothelial deposits characterized by randomly arranged large fibrils with large central bores and double layer structures (Fig. 3a, b). The mean diameter of the fibrils was 185 nm (range, 150–220 nm). These deposits resembled those described in two patients diagnosed with cryofibrinogen-associated glomerulonephritis [6]. In addition, diffuse foot process effacement and a mild increase in mesangial matrix were present. Electron dense deposits characteristic of C3 nephropathy and hump-like subepithelial deposits were not observed.

**Fig. 2:**
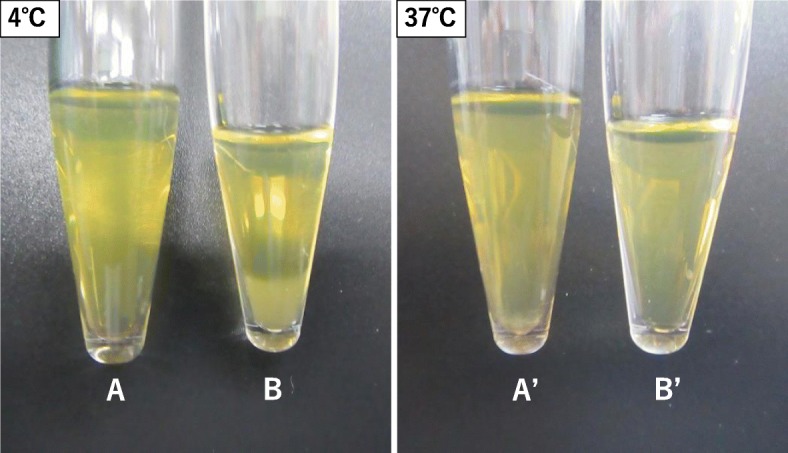
Patient’s serum (A) and EDTA plasma (B) were stored for 48 h at 4 °C, after which they were stored for 18 h at 37 °C (A, B). Cryoprotein was precipitated from plasma but not from serum. The cryoprecipitate redissolved at 37 °C

**Fig. 3:**
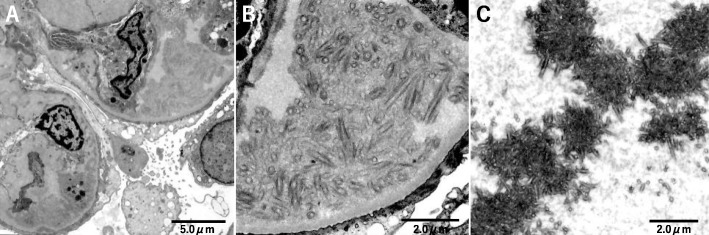
Electron microscopy. **a**, **b** Subendothelial deposits in glomeruli. Unique deposits characterized by randomly arranged large fibrils with large central bores and double layer structures are apparent. (**c**) Cryoprecipitate. Similar structures to the subendothelial deposits in glomeruli are apparent. (Original magnification, **a**: 4000×, **b**: 12,000×, **c**: 8000×)

Cryofibrinogen was detected in this patient’s serum (Fig. 2). Using prewarmed equipment, the patient’s blood was collected into both anticoagulant-free tubes and tubes containing EDTA. Both serum and plasma were prepared by centrifuging at 2000×*g* for 30 min at 37 °C. Serum and plasma were chilled to 4 °C for 48 h and analyzed for precipitation. Precipitate was found only in plasma, not in serum. When the precipitate was warmed to 37 °C, it redissolved. Ultrastructural examination of the cryoprecipitate revealed similar structures to the glomerular subendothelial deposits (Fig. 3c).

**Fig. 4:**
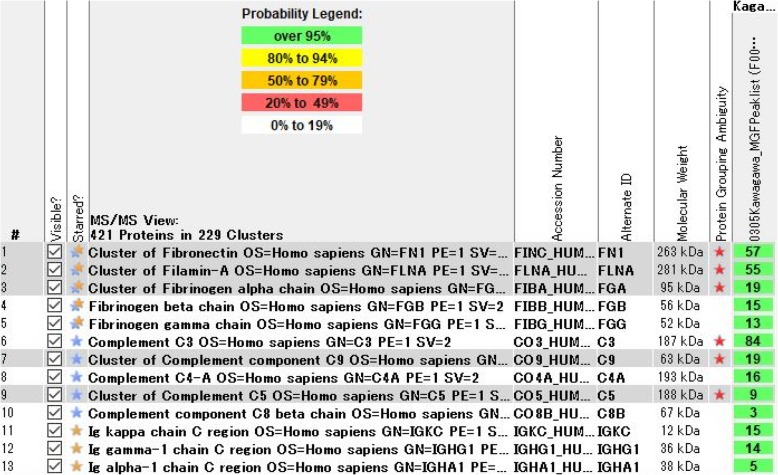
Liquid chromatography-tandem mass spectrometry (LC-MS/MS) using Mascot and Scaffold database identified increased fibrinogen α, β, and γ chains, fibronectin, filamin-A, and C3

Furthermore, LC-MS/MS on laser microdissected glomeruli from paraffin sections was performed as previously described [9], and revealed increased levels of fibrinogen α, β, and γ chains, fibronectin, filamin-A, and C3 (Fig. 4). These substances have been detected in patients previously reported as having cryofibrinogen-associated glomerulonephritis [6, 7]. IgG1, IgA1, and kappa light chain were also detected at levels comparable to those in a control. There were small amounts of proteins associated with amyloidosis such as amyloid P component and apolipoprotein A. We measured the peptide concentrations of the samples by fluorometric peptide assay (Thermo Scientific, San Jose, CA, USA) prior to LC-MS/MS analysis.

A diagnosis of cryofibrinogen-associated glomerulonephritis was made on the basis of the characteristic electron microscopic findings in the glomeruli and cryoprecipitate and results of LC-MS/MS.

**Fig. 5:**
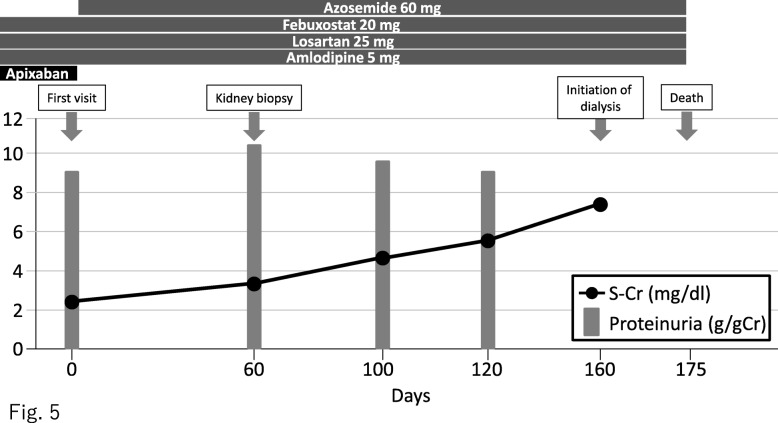
Treatment and progress of kidney function. S-Cr, serum creatinine level

After diagnosis, the patient refused to receive any additional treatment, and his renal function rapidly decreased (Fig. 5). He underwent hemodialysis 4 months after diagnosis. One month after the initiation of hemodialysis, he suddenly died, but an autopsy was not performed. Clinical data were extracted by electronic health record under the consent of the patient and patient’s family.

## Discussion and conclusions

Cryofibrinogenemia, a rare and potentially serious disorder caused by deposition of cryofibrinogen, was first described by Korst and Kratochvil in 1955 [1]. Cryofibrinogenemia may be primary or secondary. Primary cryofibrinogenemia is rare, develops spontaneously in healthy persons, and its prevalence has not yet been determined. Secondary cryofibrinogenemia is associated with various diseases, including malignancies, infections, autoimmune diseases, vasculitis, thromboembolic disease, and sepsis [3]. Reportedly associated malignancies include B-cell non-Hodgkin lymphoma, T-cell lymphoma, chronic myelomonocytic leukemia, multiple myeloma, and gastric and colorectal carcinoma [3, 10]. Reported associated infectious diseases and infective agents include *Mycobacterium tuberculosis*, *Streptococcus* spp., *Klebsiella pneumonia*, *Mycoplasma pneumoniae*, herpes zoster virus, hepatitis C virus, and Epstein–Barr virus [3, 10]. Reportedly associated autoimmune diseases include lupus, antiphospholipid syndrome, and mixed connective tissue disease. Our patient had a history of hepatitis B virus and hepatitis C virus infections (carrier status with an undetectable viral titer), gastric carcinoma (post-surgery), and non-tuberculous mycobacterial infection. Various clinical manifestations associated with cryofibrinogenemia (mainly skin conditions such as gangrene, ulceration, purpura, livedo reticularis, and Raynaud’s phenomenon) have been described. However, our patient had none of these skin conditions.

Kidney disorders associated with cryofibrinogenemia have also been reported; however, there are few published detailed descriptions of kidney pathology [4, 5]. Singh and Gaber described a membranoproliferative glomerulonephritis (MPGN) without electron-dense deposits, which they considered to represent chronic microangiopathic changes associated with cryofibrinogenemia [5]. Nash et al. described glomerular and tubular lesions characteristic of cryofibrinogenemia in a patient with type 1 diabetes mellitus [4]. Sethi et al. and Sudo et al. reported patients who had membranoproliferative glomerulonephritis with unique electron-dense deposits [6, 7]. Sethi et al. analyze cryoprecipitates using mass spectrometry. Sudo et al. performed LC-MS/MS on paraffin sections. Both groups of researchers detected fibrinogen α, β, and γ chains.

In the present case, a membranoproliferative pattern with endocapillary proliferation and crescent formation was observed by light microscopy. The capillary lumens were occluded by infiltration with inflammatory cells including neutrophils, and the capillary walls were thickened by PAS-positive subendothelial deposits. Immunofluorescence studies showed strong C3 staining and weak staining for fibrinogen along the capillary walls and mesangial areas; however, immunoglobulins were not observed except for IgM. On the basis of the light microscopic and immunofluorescence findings, we concluded that C3 nephritis or the healing stage of infection-related glomerulonephritis were possible diagnoses.

However, ultrastructural examination revealed unusual subendothelial deposits that were similar to those of the two patients reported by Sethi et al. [6]. We therefore suspected a diagnosis of cryofibrinogen-associated glomerulonephritis. Additional examination of the ultrastructure of the cryoprecipitate extracted from our patient’s plasma revealed similar structures to the glomerular subendothelial deposits. Thus, on the basis of the ultrastructural findings and LC-MS/MS results, we eventually diagnosed cryofibrinogen-associated glomerulonephritis.

In addition, no findings were found to suspect bacterial infection during the disease course; therefore, it is considered that there is no basis for the healing stage of infection-related glomerulonephritis. Because the patient’s medical history included hepatitis B virus and hepatitis C virus carrier status, gastric carcinoma, and non-tuberculous mycobacterial infection, we classified his disease as secondary based on previous reports.

However, unlike the case of Sudo et al., fibrinogen was not clearly detected by immunofluorescence despite the detection of fibrinogen by LC-MS/MS. The reason for this might be that few glomeruli were examined in the immunofluorescence studies, and the amount of fibrinogen deposited on the glomeruli was small.

Differential diagnosis of cryofibrinogen-associated glomerulonephritis includes glomerular diseases with organized deposits, including fibronectin glomerulopathy (GP), cryoglobulinemic glomerulonephritis (GN), immunotactoid GP, and fibrillary GN. According to previous reports, these can be distinguished by their immunohistochemical characteristics and details of the ultrastructure of the deposits were revealed by electron microscopy [6, 11–16]. Histological differences between these glomerular diseases are shown in Table 2. Microscopically, all these diseases often show a MPGN pattern; thus, these findings are not specific to cryofibrinogen-associated glomerulonephritis. Immunofluorescence studies are positive for IgG in cryoglobulinemic GN, immunotactoid GP, and fibrillary GN, but not in fibronectin GP. Therefore, our patient was unlikely to have cryoglobulinemic GN, immunotactoid GP, or fibrillary GN. As to the ultrastructural morphology of the deposits, cryofibrinogen-associated GN often presents as a unique, thick, double layered structure that can be differentiated from the structures found in fibronectin GP [6]. A microtubular structure can also be found in cryoglobulinemic GN and immunotactoid GP; however, these structures are thinner than those of cryofibrinogen-associated GN. These characteristics enabled us to differentiate our patient’s disorder from other diseases with organoid structures [12–16]..

**Table 2:** Pathological differentiation and characteristics of glomerular diseases with organized deposits

	Cryofibrinogen-Associated GN	Fibronectin GP	Cryoglobulinemic GN	Immunotactoid GP	Fibrillary GN
LM	MPGN	MPGN	MPGN (often with hyaline thrombi	MPGN occasionally with hyaline thrombi)	MPGN
IF (Ig deposition)	Often negative	Negative	IgG and IgM (monoclonal or oligoclonal)	IgG monoclonal or oligoclonal)	IgG (usually polyclonal)
EM					
Distribution	Subendothelial	Mesangial, subendothelial	Subendothelial	Mesangial, subepithelial, subendothelial	Mesangial, subepithelial
Appearance	Large microtubular structures	Granules with focal fibril formation	Microtubules (occasionally curved)	Microtubules	Fibrils, rarely microtubular
Size (nm)	60–211	12–16	15–45	10–90	12–24
Arrangement	Random	Random	Variable	Parallel arrays	Random

*Abbreviations: GN* glomerulonephritis, *GP* glomerulopathy, *LM* light microscopy, *IF* immunofluorescence study, *Ig* immunoglobulin, *EM* electron microscopy, *MPGN* mesangioproliferative glomerulonephritis, *IgG* immunoglobulin G, *IgM* immunoglobulin M

On the basis of our findings and those of Sethi et al., we believe that the structures that can be extracted from plasma can pass through the endothelium and form subendothelial deposits in cryofibrinogen-associated GN. The localization and size of the deposits, and the types of substances detected by LC-MS/MS may vary depending on time from onset to renal biopsy and the amount of abnormal protein in the plasma [17].

In conclusion, electron microscopic findings on kidney biopsy and plasma cryoprecipitates are crucial for the diagnosis of cryofibrinogen-associated glomerulonephritis. Although it is sometimes difficult to identify the MPGN pattern, we believe that an accurate diagnosis can be achieved by focusing on details of the clinical and pathological findings. Given that there are only a few published reports of cryofibrinogen-associated glomerulonephritis, and the mechanism by which cryofibrinogen forms characteristic structures remains unknown, further cases need to be accumulated to improve our understanding of renal involvement in patients with cryofibrinogenemia.

## Data Availability

Not applicable.

## Abbreviations

C1q: Complement 1q
C3c: Complement 3c
GFR: Glomerular filtration rate
IgA: Immunoglobulin A
IgG: Immunoglobulin G
IgM: Immunoglobulin M

## References

[1] Korst DR, Kratochvil CH (1955). Cryofibrinogen in a case of lung neoplasm associated with thrombophlebitis migrans. Blood..

[2] Stathakis NE, Karamanolis D, Koukoulis G, Tsianos E (1981). Characterization of cryofibrinogen isolated from patients plasma. Haemostasis..

[3] Saadoun D, Elalamy I, Ghillani-Dalbin P, Sene D, Delluc A, Cacoub P (2009). Cryofibrinogenemia: new insights into clinical and pathogenic features. Am J Med.

[4] Nash JW, Ross P, Neil Crowson A, Taylor J, Morales JE, Yunger TM, Magro C (2003). The histopathologic spectrum of cryofibrinogenemia in four anatomic sites. Skin, lung, muscle, and kidney. Am J Clin Pathol.

[5] Singh A, Gaber LW (2007). Nephrotic syndrome and chronic renal insufficiency associated with essential cryofibrinogenemia. Nephrol Dial Transplant.

[6] Sethi S, Yachoui R, Murray DL, Radhakrishnan J, Alexander MP (2017). Cryofibrinogen-associated glomerulonephritis. Am J Kidney Dis.

[7] Sudo M, Sakamaki Y, Hosojima M, Yamamoto S, Ito Y, Imai N (2019). Cryofibrinogen-associated glomerulonephritis diagnosed by mass spectrometry and immunoelectron microscopy. Human Pathol Case Reports.

[8] Matsuo S, Imai E, Horio M, Yasuda Y, Tomita K, Nitta K, Yamagata K (2009). Revised equations for estimated GFR from serum creatinine in Japan. Am J Kidney Dis.

[9] Aoki M, Kang D, Katayama A, Kuwahara N, Nagasaka S, Endo Y (2018). Optimal conditions and the advantages of using laser microdissection and liquid chromatography tandem mass spectrometry for diagnosing renal amyloidosis. Clin Exp Nephrol.

[10] Belizna CC, Tron F, Joly P, Godin M, Hamidou M, Levesque H (2008). Outcome of essential cryofibrinogenaemia in a series of 61 patients. Rheumatology (Oxford, England).

[11] Lusco MA, Chen YP, Cheng H, Dong HR, Najafian B, Alpers CE (2017). AJKD atlas of renal pathology: Fibronectin Glomerulopathy. Am J Kidney Dis.

[12] Herrera GA, Ojemakinde KO, Turbat-Herrera EA, Gu X, Zeng X, Iskandar SS (2015). Immunotactoid Glomerulopathy and Cryoglobulinemic nephropathy: two entities with many similarities. A Unified Conceptual Approach. Ultrastructural Pathol.

[13] Nasr SH, Fidler ME, Cornell LD, Leung N, Cosio FG, Sheikh SS (2012). Immunotactoid glomerulopathy: clinicopathologic and proteomic study. Nephrol Dialysis Transplantation.

[14] Bridoux F, Hugue V, Coldefy O, Goujon JM, Bauwens M, Sechet A (2002). Fibrillary glomerulonephritis and immunotactoid (microtubular) glomerulopathy are associated with distinct immunologic features. Kidney Int.

[15] Rosenstock JL, Markowitz GS, Valeri AM, Sacchi G, Appel GB, D'Agati VD (2003). Fibrillary and immunotactoid glomerulonephritis: distinct entities with different clinical and pathologic features. Kidney Int.

[16] Alpers CE, Kowalewska J (2008). Fibrillary glomerulonephritis and immunotactoid glomerulopathy. J Am Soc Nephrol.

[17] Elema JD, Hoyer JR, Vernier RL (1976). The glomerular mesangium: uptake and transport of intravenously injected colloidal carbon in rats. Kidney Int.

